# Association of Fat Mass and Skeletal Muscle Mass with Cardiometabolic Risk Varied in Distinct PCOS Subtypes: A Propensity Score-Matched Case-Control Study

**DOI:** 10.3390/jcm13020483

**Published:** 2024-01-15

**Authors:** Jie Cai, Jiang Yue, Nan Lu, Shengxian Li, Jun Zheng, Rong Huang, Yihong Jiang, Chang Shan, Wei Liu, Jing Ma, Lihua Wang

**Affiliations:** Department of Endocrinology and Metabolism, Renji Hospital, School of Medicine, Shanghai Jiao Tong University, Shanghai 200127, China; caijie016409@renji.com (J.C.); yuejiang@renji.com (J.Y.); lunan411@163.com (N.L.); rj_shengxianli@163.com (S.L.); 8575@renji.com (J.Z.); jiangyihong@renji.com (Y.J.); danchang@renji.com (C.S.); sue_liuwei@163.com (W.L.)

**Keywords:** fat mass, skeletal muscle mass, cardiometabolic risk, PCOS, obesity

## Abstract

(1) Background: polycystic ovarian syndrome (PCOS) is a heterogeneous syndrome with a constellation of cardiometabolic risk factors. We aimed to investigate if the association of body fat mass (BFM) and skeletal muscle mass (SMM) with cardiometabolic risk differed in PCOS subtypes. (2) Methods: 401 participants (245 PCOS and 156 controls) were assessed for anthropometric measurements, glucose–lipid profiles, reproductive hormones and body composition with propensity score-matched (PSM) analysis. The association of the cardiometabolic risk score (z score, calculated based on levels of obesity and gluco-lipid measurements) with BFM (estimated by trunk BFM/Height^2^) and SMM (estimated by SMM/Height^2^) was calculated. (3) Results: Trunk BFM/Height^2^ and SMM/Height^2^ were both positively associated with cardiometabolic risk in PCOS (trunk BFM/Height^2^, OR 2.33, 95% CI 1.49–3.65; SMM/Height^2^, OR 2.05, 95% CI 1.12–3.76). SMM/Height^2^ associated with increased cardiometabolic risk in obese PCOS (BMI ≥ 28 kg/m^2^, OR 2.27, 95% CI 1.15–4.47). For those with lower BMI (<28 kg/m^2^), trunk BFM/Height^2^ showed a higher OR in both groups (PCOS, OR 2.12, 95% CI 1.06–4.24; control 2.04, 95% CI 1.04–4.02). Moreover, distinct associations among BMI-stratified groups were validated in hierarchical clustering identifying metabolic and reproductive clusters. (4) Conclusions: BFM and SMM are synergistically associated with higher cardiometabolic risk in PCOS women. Although BFM contributes to increased cardiometabolic risk, SMM also plays a primary role in obese PCOS. Our results highlight the importance of body composition in the management of PCOS.

## 1. Introduction

Women with polycystic ovary syndrome (PCOS) are predisposed to cardiometabolic comorbidities such as prediabetes/type 2 diabetes mellitus (T2DM), dyslipidemia, obesity, metabolic syndrome, and cardiovascular events [1,2,3,4,5,6,7]. Insulin resistance is considered to be the principal factor in the pathogenesis of PCOS and is related to an increased level of cardiometabolic risk factors [8,9,10,11].

Insulin resistance has linked an altered body composition with cardiometabolic disturbance in PCOS. Adipose tissue and skeletal muscle tissue are two main target tissues for insulin-stimulated glucose disposal [12,13]. Excessive android fat mass (trunk and upper body fat mass) accumulation has been reported to be associated with cardiometabolic disturbance in PCOS [14,15,16,17,18]. This concept is also supported in non-obese PCOS women. As approximately 30–50% of PCOS patients with normal body weight have insulin resistance, they also have higher cardiometabolic risks [9]. Therefore, to the best of our knowledge, studies regarding this are limited study in non-obese PCOS women and provide little information on their skeletal muscle simultaneously. Skeletal muscle accounts for the majority of insulin-stimulated glucose uptake in vivo [19]. Insulin resistance by defective insulin signaling at receptors and/or postbinding levels altered the muscle glucose uptake of PCOS [20,21,22,23,24]. However, contradictory evidence regarding the skeletal muscle mass in PCOS women has been reported. Some studies found reduced total or appendicular muscle mass in PCOS compared to control women. On the other hand, some studies reported no significant difference between PCOS and controls. Moreover, there is also evidence to show increased muscle mass or strength in PCOS [25,26,27,28]. These conflicting findings might be derived from the small sample size, the different diagnostic criteria applied or undefined PCOS subtypes. The clinical presentation of PCOS is heterogeneous, and the etiologies in PCOS is unknown even with decades of effort. Genetic studies have reported genetic association on several pathways such as metabolic regulation, androgen biosynthesis, and gonadotropin secretion and action [29], suggesting physiological diversity in PCOS. So the long-term complications (such as cardiometabolic risk) might also possibly be varied in different PCOS subtypes. A recent study applied unsupervised clustering to subtype PCOS by quantitative clinical traits, and revealed a distinct genetic architecture in biologically relevant PCOS subtypes [29], which provides a promising method other than existing diagnostic criteria to better classify subtypes in PCOS. However, whether fat mass and skeletal muscle mass act differently among distinct PCOS subtypes is unknown. In the present study, we aimed to investigate the association of fat mass and skeletal muscle mass with cardiometabolic risk in PCOS subtypes and control women matched for age, BMI, and WC by using propensity score matching (PSM).

## 2. Materials and Methods

### 2.1. Study Design

**Figure 1:**
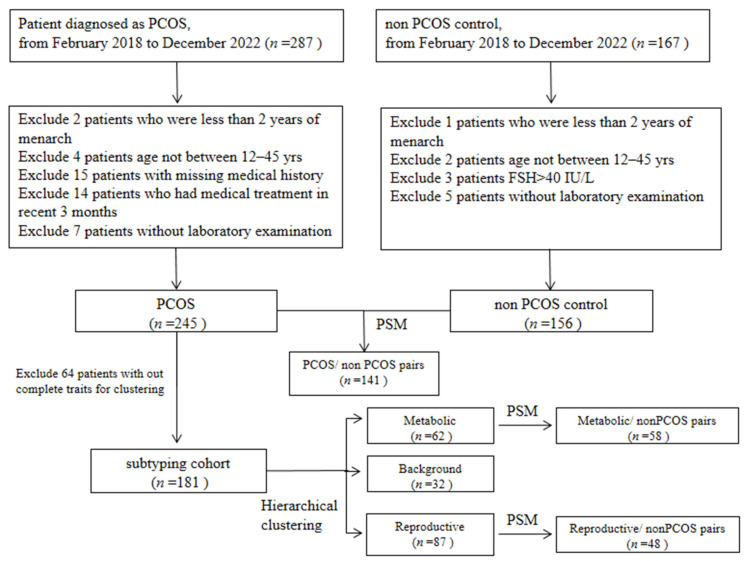
Flow diagram of the population included in the study.

Flow chart of this study is shown in Figure 1. This propensity score-matched case–control study was performed from February 2018 to December 2022 in the Department of Endocrinology, Renji Hospital, Shanghai. Investigators received training relative to the study questionnaire and outcome measures before conducting the investigation.

### 2.2. Study Participants

In total, 401 individuals (245 PCOS and 156 non-PCOS controls) fulfilled the inclusion criteria and were finally included in the study. PCOS was diagnosed according to Rotterdam criteria 2003 with at least two of the three following aspects: (1) oligo/amenorrhea/anovulation, (2) hyperandrogenemia or clinical manifestations of high blood androgen, and (3) PCO morphology. Oligomenorrhea was defined as an intermenstrual interval of 35 days and less than 8 menstrual bleedings in the past year. Amenorrhea was defined as absent menstrual bleeding or none in the past 90 days. Hyperandrogenemia is defined as total testosterone (T) > 2.6 mmol/L or an FAI (free androgen index) > 7. Hirsutism is assessed by the modified Ferriman–Gallwey (mF-G) score ≥ 4 for the recommendation of Chinese women [30]. Acne was evaluated using the Comprehensive Acne Severity Scale (CASS) [31]. PCO morphology was defined by ultrasound findings of polycystic ovaries in one or two ovaries or ≥12 follicles measuring 2–9 mm in diameter and/or an ovarian volume ≥ 10 mL in either side. The PCOS women were all negative for endocrine disease, such as nonclassical 21-hydroxylase deficiency, Cushing’s syndrome, androgen-secreting tumors, hyperprolactinemia or thyroid dysfunction. Subjects in the control group were healthy women with normal ovulation cycles and without clinical or biochemical HA. Exclusion criteria for PCOS and control women were being under the treatment of oral contraceptives or any medications that could affect hormone profiles or glucose hemostasis (e.g., anti-androgens, insulin, metformin, insulin sensitizers, and glucocorticoids) within the past 3 months. We also limited our participants to at least two years after menarche and within 45 years. Written consent was obtained from all participants. The study was conducted in accordance with the Declaration of Helsinki and approved by the Ethical Review Board of Renji Hospital ((2015)037).

### 2.3. Data Collection and Measurements

After 12 h of fasting, blood samples were collected between days 2 and 5 after spontaneous or progestogen withdrawal bleeding episodes in PCOS and control women. Triglycerides (TG), total cholesterol (TC), low-density lipoprotein cholesterol (LDL), high-density lipoprotein cholesterol (HDL), blood glucose (FBG), serum creatinine and γ-glutamyl transferase (γ-GT) were analyzed by Roche/Hitachi analyzers using Roche reagents (D 2400 and E 170 Modular Analytics modules; Roche Diagnostics, Indianapolis, IN, USA). Serum follicle-stimulating hormone (FSH), luteinizing hormone (LH), testosterone (T), sex hormone binding globulin (SHBG), DHAES, androstenedione (A2) and serum insulin levels were measured using chemiluminescence (Elecsys Auto analyzer, Roche Diagnostics). The free androgen index (FAI) was determined as follows: FAI = total testosterone (nmol/L) × 100/SHBG (nmol/L). BMI was calculated using the following formula: BMI = weight (kg)/height (m^2^). A homeostasis model assessment of insulin resistance (HOMA-IR) was calculated by the following formula: HOMA−IR = (fasting insulin [mIU/L] × fasting glucose [mmol/L])/22.5). The Matsuda Index using 0 and 120 min glucose and insulin levels [32] were taken from an online calculator at this website (http://mmatsuda.diabetes-smc.jp/2points.html accessed on 4 February 2023).

### 2.4. Body Composition Assessment

Body composition indices were assessed by bioelectrical impedance analysis (BIA) using multifrequency-based proprietary algorithms (InBody 770; Biospace, Seoul, Republic of Korea). BIA is an inexpensive and noninvasive method for estimating body composition compartments without the risk of radiation exposure for reproductive women [33,34]. Participants were asked to assess body composition after their menstrual period in the morning, 4 h after drinking water and having urinated before assessment. The data obtained included total and segmental body composition parameters: appendicular (limb extremities), trunk or total body fat mass (BFM) or skeletal muscle mass (SMM) adjusted by height^2^ or BMI, percent of body fat (PBF), and visceral fat area.

### 2.5. Main Outcome and Calculation of the Cardiometabolic Risk Score

The main outcome of this study is higher cardiometabolic risk. We dichotomized each group by their group-specific cardiometabolic risk score, and higher cardiometabolic risk was defined as the upper cardiometabolic risk score. Clustered risk factors related to metabolic health in PCOS were included [2]: obesity (BMI, WC), insulin resistance (log-transformed fasting and 2 h insulin), hyperglycemia (log-transformed fasting and 2 h plasma glucose), and dyslipidemia (LDL, inverted fasting HDL cholesterol multiplied −1, and log-transformed triglycerides). The blood pressure level was not included since there has been inconclusive results on hypertension in adolescents or women of reproductive age with PCOS [2]. The cardiometabolic risk score was derived by standardizing and then summing these continuously distributed indices of the z scores of these factors and then dividing them by the number of risk factors to compile the cardiometabolic risk score, with units of SD [35,36]. The mean of this continuously distributed cardiometabolic risk score is therefore zero by definition. The cardiometabolic risk score was identified in a confirmatory factor analysis and shown across the age spectrum in adults [36]. The purpose of using a continuously distributed variable was to maximize statistical power [37,38].

### 2.6. Statistics

All data are expressed as mean ± SD for continuous variables with a normal distribution or median (IQR) for continuous variables with a skewed distribution. Student’s *t*-tests or Wilcoxon rank-sum tests were used to compare two groups of continuous variables. Body composite indices representing fat mass or skeletal muscle mass or adjusted by Height^2^ or BMI were analyzed by a linear regression model to assess the proportion of the variance explained (R^2^) of cardiometabolic risk in the specified group. The best-performing fat mass or skeletal muscle mass indices (trunk BFM/Height^2^ and SMM/Height^2^, respectively) were used in the analysis in our study (Table S1). Data were double checked by two independent investigators.

Tertiles of fat mass or skeletal muscle mass were defined by group-specific cutoffs in each subgroup among all participants and subgroups separately (Table S2). Logistic regression was used to analyze the association of fat mass or skeletal muscle mass with higher cardiometabolic risk. ORs (95% CIs) were adjusted by age, body weight, serum creatinine and γ-glutamyl transferase; fat mass or skeletal muscle mass were mutually adjusted. We also analyzed the joint associations of obesity on the main associations of fat mass or skeletal muscle mass with higher cardiometabolic risk. A BMI over 28 kg/m^2^ was applied to identify obesity, as has been recommended as the cutoff value in the Chinese population [39]. The effect of multiplicative interactions between obesity and fat mass/skeletal muscle mass on higher cardiometabolic risk was analyzed by including the respective product term (BMI × body composition indices) in the models among all participants and subgroups separately. Analysis of variance (ANOVA) or Kruskal–Wallis one-way analysis of variance by ranks was performed among groups divided by group-specific fat mass or skeletal muscle mass tertiles.

The propensity score was determined based on logistic regression models, and the matching tolerance obtained by matching was evaluated by calculating absolute standardized differences in covariates within the two groups [40]. Briefly, propensity score-matched (PSM) analysis was used to further control for potential confounders (age, BMI, and waist circumference) between the total/subtyped PCOS women and controls, with a caliper of 0.03.

To analyze whether the association pattern of body composite varies in distinct subtypes of PCOS women, we applied an unsupervised, agglomerate, hierarchical clustering as previously described [29]. Briefly, eight traits (BMI, fasting glucose and insulin, LH, FSH, T, DHEAS and SHBG) were included in this analysis. Quantitative trait values were then log-normalized and adjusted for age and assay method. Cluster stability indicated by Jaccard coefficients above 0.6 suggests that the cluster reflects a real pattern within the data.

Statistical significance was analyzed using a two-sided *p* value of <0.05. The PCOS subtyping cluster was conducted by R software (version 4.2.2). All other statistical analysis was performed using SPSS software (version 27.0, SPSS Inc., Chicago, IL, USA).

## 3. Results

### 3.1. Characteristics of Body Composite Indices, Anthropometric and Metabolic Measures, and Reproductive Hormones in PCOS and Non-PCOS Women

Table 1 illustrates the anthropometric, hormonal and metabolic characteristics in participants with/without PCOS. As trunk BFM/Height^2^ and SMM/Height^2^ showed the best performing indices to assess cardiometabolic risk in PCOS and the controls, we used them as surrogates of fat mass or skeletal muscle mass in the following analysis. Before PSM, PCOS women had a significantly lower visceral fat area (VFA), body fat percentage (PBF), trunk BFM/Height^2^ and SMM/Height^2^ than the controls (all *p* < 0.05). Additionally, they had a lower age, BMI and WC than the non-PCOS groups (all *p* < 0.001). The prevalence of obesity (BMI ≥ 28 kg/m^2^) was lower in the PCOS group before PSM. To balance age, BMI and WC levels, we applied PSM with a 1:1 ratio, and 141 PCOS and control pairs were subsequently analyzed. After PSM, trunk BFM/Height^2^ and SMM/Height^2^ and other cardiometabolic risk factors (glucose and lipid profiles) were comparable between the two groups. PCOS women have higher levels of postprandial 2 h insulin and a lower Matsuda index and SHBG compared to their counterparts (all *p* values < 0.05).

### 3.2. Association between Trunk BFM/Height^2^ Levels and Cardiometabolic Risk in PCOS and Controls

**Figure 2:**
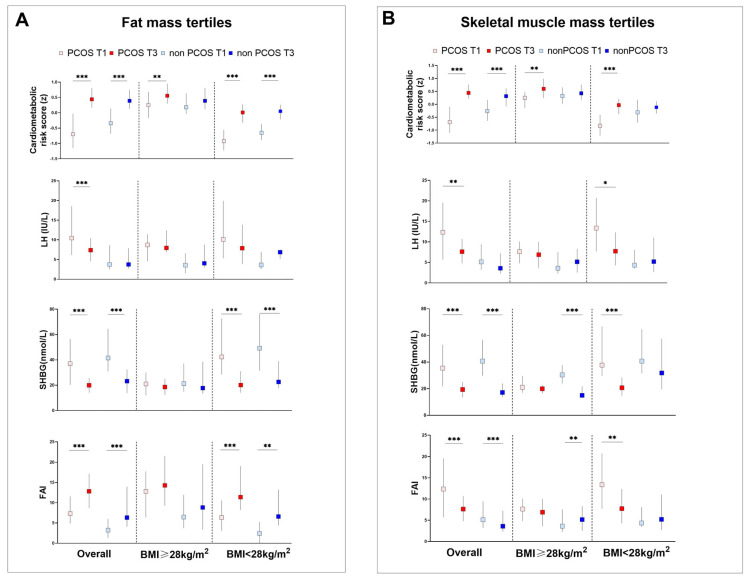
Cardiometabolic risk score and reproductive profiles by trunk BFM/Ht^2^ and SMM/Ht^2^ levels stratified by BMI. Cardiometabolic risk scores and hormone levels in tertiles of (**A**) trunk BFM/Ht^2^ and (**B**) SMM/Ht^2^ among all participants and among participants stratified by BMI. Tertiles of trunk body fat mass (BFM) adjusted by height square (Trunk BFM/Ht^2^) and skeletal muscle mass (SMM) adjusted by height square (SMM/Ht^2^) were group-specific in each subgroup; error bars indicate IQR in each traits. A *p* value between a higher tertile (upper tertile, T3) and lower tertile (lower tertile, T1) was marked with asterisk (* <0.05, ** <0.01 and *** <0.001); otherwise, they were not significant.

A higher trunk BFM/Height^2^ was associated with metabolic (increased BMI, WC, glucose/insulin, lipid profiles, and cardiometabolic risk score) and reproductive (increased FAI and SMM/Height^2^ and decreased SHBG and LH/FSH levels) factors (Figure 2A and Table S3) among overall PCOS and control participants. Those belonging to the upper tertile of the trunk BFM/Height^2^ measure had increased LDL and decreased LH in the PCOS group but they were comparable in controls. In the BMI-stratified analysis, in obese (BMI ≥ 28 kg/m^2^) PCOS, a higher trunk BFM/Height^2^ level was associated with a higher cardiometabolic risk score than in control women. In individuals with a lower BMI (<28 kg/m^2^), a higher trunk BFM/Height^2^ level is associated with cardiometabolic risk factors (BMI, WC, fasting/2 h postprandial glucose or insulin, and lipid profiles) in the PCOS group (*p* for trend < 0.05). Meanwhile, in the control group, less factors were associated with higher trunk BFM/Height^2^ levels. 

**Figure 3:**
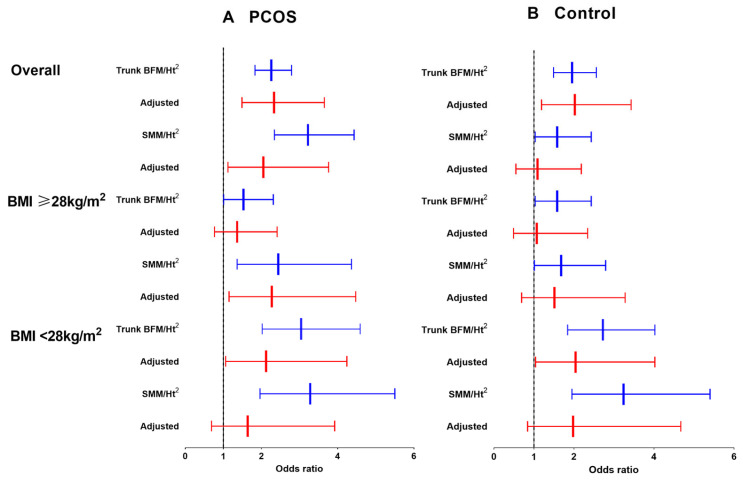
Association between trunk BFM/Ht^2^ or SMM/Ht^2^ with higher cardiometabolic risk among PCOS and control participants by BMI categories. Association between trunk BFM/Ht^2^ and SMM/Ht^2^ with higher cardiometabolic risk among (**A**) PCOS and (**B**) control women by BMI categories divided by BMI (≥28 kg/m^2^ or <28 kg/m^2^). Trunk BFM adjusted by height square (trunk BFM/Ht^2^) and SMM adjusted by height square (SMM/Ht^2^) tertiles were group-specific among each subgroup; error bars indicate 95% CIs. ORs (95% CIs) were adjusted for age, body weight, serum creatinine and γ-glutamyl transferase; trunk BFM/Ht^2^ and SMM/Ht^2^ were mutually adjusted. BFM, body fat mass; SMM, skeletal muscle mass. Blue, raw ORs; Red, adjusted ORs.

We then investigated the association between the trunk BFM/Height^2^ with a higher cardiometabolic risk in the overall and BMI-stratified PCOS. For the overall cohort, a higher trunk BFM/Height^2^ was associated with increased cardiometabolic risk after adjusting for SMM/Height^2^, age, body weight, serum creatinine and γ-glutamyl transferase (PCOS: OR, 2.33; 95% CI 1.49–3.65; control: OR, 2.02; 95% CI 1.19–3.43. Table 2 and Figure 3A). To investigate whether obesity modified fat mass and skeletal mass in association with higher cardiometabolic risk, we further analyzed the interaction effects of BMI and trunk BFM/Height^2^ (*p*_interaction_ < 0.001 in PCOS; *p*_interaction_ < 0.001 in control), BMI and SMM/Height^2^ (*p*_interaction_ < 0.001 in PCOS; *p*_interaction_ < 0.001 in control). Then, we divided each group by BMI ≥ 28 kg/m^2^, which is recommended as a BMI cutoff in the Chinese population for obesity. Trunk BFM/Height^2^ did not show a significant association with cardiometabolic risk in either group. However, in the nonobese (BMI < 28 kg/m^2^) group, a positive association was observed in both PCOS and the controls (PCOS: OR, 2.12; 95% CI 1.06–4.24; control: OR, 2.04; 95% CI 1.04–4.02). Increased trunk BFM/Height^2^ tertiles were also associated with increased SMM/Height^2^ in the overall and BMI-stratified analysis, respectively.

### 3.3. Association between SMM/Height^2^ Levels and Cardiometabolic Risk in PCOS and Controls 

Figure 2B and Table S4 show cardiometabolic risk factors and reproductive profiles in PCOS and controls by SMM/Height^2^ tertiles. For overall individuals, more variables of cardiometabolic risk factors increased with SMM/Height^2^ tertiles in PCOS. For reproductive hormones, although FAI increased and SHBG levels decreased with higher SMM/Height^2^ tertiles in both groups, we observed a decreased LH and LH/FSH only in the PCOS group by higher SMM/Height^2^ tertiles. In the BMI-stratified analysis, similar patterns of cardiometabolic risk factors was observed in nonobese individuals except the association of reproductive hormones was distinct among PCOS and controls. In obese individuals, lower SHBG and higher FAI were observed only in controls by increased SMM/Height^2^ tertiles. However, in nonobese women, this pattern was observed in PCOS instead, with decreased LH and LH/FSH as well (Table S4).

The link between SMM/Height^2^ and cardiometabolic risk in the overall and BMI-stratified subgroups is illustrated in Table 2 and Figure 2B. SMM/Height^2^ and trunk BFM/Height^2^ synergistic associated with higher cardiometabolic risk in overall PCOS women (OR, 2.05; 95% CI 1.12–3.76). Higher SMM/Height^2^ is associated with higher cardiometabolic risk only in obese PCOS women after adjusting for trunk BFM/Height^2^, age, body weight, serum creatinine and γ-glutamyl transferase (OR, 2.27; 95% CI 1.15–4.47) (Figure 2B and Table 2). However, in the nonobese subgroup, there was no association between SMM/Height^2^ and cardiometabolic risk in both groups.

### 3.4. Body Composition, and Metabolic and Reproductive Hormones in Participants with Obesity and HA 

Obesity could increase functional circulating androgens by decreasing hepatic SHBG secretion. To further explore the unique association of SMM/Height^2^ levels with cardiometabolic risk among obese PCOS, we compared body composition, and metabolic and reproductive hormones in PCOS and control women with obesity and HA (Table 3). Despite comparable androgens (T, FAI, DHEAS and A2) and body composite indices (trunk BFM/Height^2^ and SMM/Height^2^), PCOS women had lower WC and higher SHBG than controls (all *p* < 0.05).

### 3.5. Characteristics of Anthropometric Measures, Metabolic and Reproductive Hormones and Body Composite Indices in PCOS Subtypes and Control Women

As shown in Figures S1–S3, 181 PCOS women were clustered into three phenotypic groups: a metabolic subtype (*n* = 62), characterized by higher BMI, fasting glucose and fasting insulin levels; a reproductive subtype (*n* = 87), characterized by higher LH and SHBG and relatively lower BMI, glucose and insulin levels; and the remaining cases, designated the indeterminate cluster (*n* = 32), as they demonstrated no distinguishable clinical trait pattern. The Jaccard coefficients in the metabolic, reproductive and indeterminate clusters were 0.66, 0.63 and 0.4, respectively.

Body composition indices, and anthropometric and metabolic/hormonal characteristics before and after PSM are illustrated in Table S5 and Table 4. The metabolic subtype was associated with increased body composite indices (trunk BFM/Height^2^ and SMM/Height^2^), cardiometabolic risk score and risk factors (BMI, WC, fasting and postprandial glucose and insulin, HOMA IR, and lipid profiles), reproductive hormones (LH, FSH, FAI, and DHEAS), and a lower Matsuda index and SHBG than the reproductive subtype (Table S5). The PCA plot (Figure S3) also illustrated a similar axis direction as previously reported in the European cohort [29]. Similar association patterns from the above BMI-stratified analysis were validated among unsupervised clustering subtypes. We compared the differences between distinct PCOS subtypes with age, BMI, and WC-matched control women by applying 1:1 PSM in each subtype. After PSM (Table 4), the metabolic subtype had significantly increased trunk BFM/Height^2^ and SMM/Height^2^ levels and a higher cardiometabolic risk score compared with controls (all *p* < 0.05). SMM/Height^2^ showed a positive association with cardiometabolic risk in metabolic PCOS (BMI, 30.9 ± 4.8 kg/m^2^) (OR 2.63, 95% CI 1.13–6.16). However, in the reproductive subtype, the body composite indices and cardiometabolic risk score were similar compared to their counterparts. Reproductive PCOS (BMI, 22.8 ± 4.2 kg/m^2^) showed a positive association between trunk BFM/Height^2^ and cardiometabolic risk (OR 2.37, 95% CI 1.33–4.21) (Table 5).

## 4. Discussion

In the present study, we found an association between body composition and cardiometabolic risk differences in distinct subtypes of PCOS. Fat mass, estimated using trunk BFM/Height^2^, was associated with a higher cardiometabolic risk both in PCOS and control women. Skeletal muscle mass, estimated using SMM/Height^2^, has a positive association with higher cardiometabolic risk in obese PCOS women. Additionally, our results in hierarchical clustering-subtyped PCOS women provide a new understanding of body composition among distinct subtypes of PCOS. 

Body composition indices based on total or segmental fat/muscle mass, associated with a higher risk of metabolic diseases, have been reported. However, there are no uniform body composition indices applied to all ethics, age or diet styles to date. Fat free mass (FFM) divided by height^2^ was related to metabolic syndrome risk only in females. A lower body skeletal muscle or skeletal muscle index (SMI, appendicular skeletal muscle divided by height^2^) indicates worse glucose control and severe insulin resistance in T2DM and sarcopenia [15,41]. We used the trunk BFM/Height^2^ and whole-body SMM/Height^2^ as they demonstrated the best performance in the cardiometabolic risk score. A previous study on nutritional assessment suggested that height-normalized body composition indices are useful to overcome nutrition status among individuals [42]. Our participants were young, overweight/obese women (BMI, PCOS, 26.66 ± 5.71 kg/m^2^; control, 29.11 ± 5.91 kg/m^2^). Therefore, our results are consistent with a meta-analysis showing that PCOS women with a BMI greater than 25 kg/m^2^ have greater total lean body mass or fat-free mass [21]. After PSM, metabolic PCOS had higher SMM/Height^2^ levels than the controls (PCOS vs. control: 9.98 ± 1.06 vs. 9.39 ± 1.38 kg/m^2^). And SMM/Height^2^ is also associated with higher cardiometabolic risk in the metabolic PCOS subtype.

Fat tissue and skeletal muscle are major determinants of insulin-mediated glucose uptake [19]. Insulin resistance via a postbinding defect at an early stage in receptor signaling links PCOS to an increased risk of prediabetes and T2DM later in life [9,10,11,43,44,45,46,47,48]. Our results show that in PCOS individuals, the highest tertiles of trunk BFM/Height^2^ and SMM/Height^2^ are associated with more cardiometabolic risk factors than in the controls (Tables S3 and S4). This suggests that intrinsic insulin resistance in PCOS may affect both fat and skeletal muscle. In addition to glucose hemostasis dysfunction, PCOS women exhibit a constellation of traits related to cardiometabolic risk factors such as central obesity, dyslipidemia, nonalcoholic fatty liver disease (NAFLD), HA, hypertension, and CVD [49]. An excessive distribution of android fat with higher cardiometabolic risk has been established in PCOS [15,50,51,52,53]. Adipocytes also produce excessive inflammatory cytokines such as tumor necrosis factor-alpha (TNF-a), interleukin 6 (IL6), leptin, and visfatin, which are associated with obesity, IR, or CVD [2]. Androgens play an important role as a regulator of muscle protein synthesis and muscle hypertrophy. It can increase the size and strength of skeletal muscle across young and old individuals, with an increased cross-sectional area of both type I and II muscle fibers [54,55]. Androgens’ anabolic action can established through Akt, myostatin, IGF-1 or Notch signaling, and result in the stimulation of protein synthesis and inhibition of degradation [55,56]. In PCOS, an irregular pulse of the hypothalamic–pituitary–ovarian (HPO) axis stimulates ovarian androgen secretion and inhibits hepatic SHBG production [10]. In control women, HA is often attributed to obesity, as abdominal fat distribution could reduce SHBG synthesis and contribute to a higher level of FAI, a bioactive androgen form [57]. Thus, a lower SHBG level, subsequent to insulin resistance, could also contribute to HA and is associated with increased adverse metabolic profiles and hypertension [2,57]. Our result is in accordance with the above studies: PCOS women have relatively higher LH and SHBG as well as lower WC than controls, despite the similar androgen levels between PCOS and the controls with both obesity and HA. These results suggested that HA in control women might be attributed to central fat distribution instead of ovarian androgen secretion. Similar results were found in the metabolic subtype of PCOS women, as they had prominently elevated LH, LH/FSH, and androgens, lower SHBG and a higher cardiometabolic risk score after PSM. These results indicate that HA subsequent to activation of the HPO axis could amplify the susceptibility to develop cardiometabolic abnormities in PCOS with obesity.

Excess body fat is linked to intermuscular (between muscle fascicles/cells) and intramuscular (within muscle cells) fat infiltration, which is recognized as a risk for unfavorable cardiometabolic health by secretion of adipokines and metabolites [58,59]. Higher intramuscular adipose tissue (IMAT) is associated with increased all-cause mortality and cardiovascular disease mortality after adjusting for trunk fat mass in men [59]. Our previous study showed increased thigh muscle IMAT accessed by MRI was associated with glucose dysregulation in the obese population [60]. In women with PCOS, higher thigh IMAT has been reported to be associated with increased insulin resistance [61]. The excess fat storage in muscle could deleteriously reduce muscle strength and contribute to sarcopenia. However, a generally accepted definition and common diagnostic criteria is still on the way [62]. So we were not able to evaluate the association in sarcopenic women in our study. We applied BIA to assess fat mass and skeletal muscle mass for its advantages in terms of being easy, cost-efficient and radiation-free. However, BIA is unable to accurately differentiate intramuscular fat from muscle mass, which could lead to an overestimate of muscle mass in the obese population. In contrast, magnetic resonance imaging (MRI) is feasible for quantitatively assessing intramuscular lipid content, although it is time-consuming, costly and requires technical expertise. In a correlation study on skeletal muscle mass assessment by MRI and BIA, MRI might outperform BIA in the quantification of skeletal muscle mass parameters, specifically in obese subjects with a BMI over 30 kg/m^2^ [63]. Given the limitations of the BIA method, the positive association between skeletal muscle mass and higher cardiometabolic risk in obese PCOS should be interpreted with caution, as increased IMAT might, at least partially, account for skeletal muscle mass. Further studies are needed to evaluate ectopic lipid accumulation among obese PCOS women using more sophisticated methods, such as MRI.

Body weight reduction is well accepted as an important part of the treatment of cardiometabolic diseases. Studies have shown that 21–50% of adolescents and young adults with PCOS are overweight or obese [2]. Interventions of weight loss have consistently been linked to reductions in cardiovascular risk factors, such as improved glycemic control, insulin sensitivity, and cardiorespiratory fitness [64,65]. Various strategies focused on body composition have been employed to achieve body weight loss in obese PCOS women. A reduction in VAT through calorie-restrictive therapy has shown promising effects on cardiometabolic risk in obese PCOS women [66]. Having undergone bariatric surgery, those with a lower final BMI were associated with complete remission of PCOS (defined as six consecutive regular menstruation cycles or spontaneous pregnancy) [67]. Decreased body fat percentage and increased lean body mass are associated with decreased testosterone and glucose levels after 4 months of resistance training in PCOS [68]. However, these results were constrained by the small sample size and lack of analysis by BMI stratification. We found a distinct pattern in body composition with cardiometabolic risk among PCOS and controls. Understanding these unique patterns between PCOS and controls, as well as between obese and nonobese PCOS individuals, could greatly assist in determining specific and dynamic lifestyle intervention strategies. Fat mass plays a key role in cardiometabolic risk in the control group and nonobese PCOS women, as expected. Trunk BFM/Height^2^ and SMM/Height^2^ were synergistically associated with a higher OR of cardiometabolic risk in overall PCOS. In obese PCOS, SMM/Height^2^ was associated with a higher OR, indicating obesity might modify the association of BFM and SMM with cardiometabolic risk in a distinct way among PCOS and controls (Table 2 and Figure 3). Considering the synergistic effect of BFM and SMM in PCOS, emphasis should be placed on BMI reduction, especially in obese PCOS. On the other hand, resistance exercise combined with weight loss could be recommended when PCOS women achieve a relatively lower BMI level. There are distinct subtypes of PCOS, so individualized strategies should be applied to manage specific subtypes of patients. These results also highlight that body composition might be useful to subtype PCOS with high cardiometabolic risk.

## 5. Limitations

The present study had several limitations. First, this is a cross-sectional study, and we were not able to draw causal conclusions. Second, we adjusted for multiple confounders that may have affected fat mass and skeletal muscle mass and used indices adjusted by height^2^ to overcome malnutrition. However, bias from unmeasured confounding factors likely exists. Third, we assessed the quantity of fat mass and skeletal muscle mass with cardiometabolic risk among young women. Future studies are needed to validate our findings with larger sample sizes across age and ethnicities in longitudinal studies. Our results could also be clarified in sarcopenic patients once a generally accepted definition and diagnostic criteria are established. Fourth, the association between skeletal muscle mass and higher cardiometabolic risk in obese PCOS by BIA should be interpreted with caution, as increased IMAT might account for skeletal muscle mass. Future studies using more sophisticated methods, such as MRI-based technology, could provide additional evidence.

## 6. Conclusions

This propensity score-matched case–control study showed that trunk BFM/Height^2^ and SMM/Height^2^ are synergistically associated with higher cardiometabolic risk in patients with PCOS. Although fat mass is the key body composition contributing to higher cardiometabolic risk, there was a unique association of skeletal muscle mass among obese women with PCOS. Our results highlight the importance of both fat mass and skeletal muscle mass in the management of PCOS to reduce their cardiometabolic risk.

## Figures and Tables

**Table 1:** Clinical characteristics and body composite indices before and after propensity score-matched women with or without PCOS.

	Before PSM			After PSM		
	PCOS (*n* = 245)	Control (*n* = 156)	*p* Value ^(1)^	PCOS (*n* = 141)	Control (*n* = 141)	*p* Value ^(2)^
Age (years)	28 ± 6	31 ± 7	<0.001	30 ± 6	30 ± 7	0.992
BMI (kg/m^2^)	26.7 ± 5.7	29.1 ± 5.9	<0.001	28.5 ± 5.7	28.5 ± 5.3	0.941
BMI ≥ 28 kg/m^2^ (%, *n*)	40.0 (98)	53.8 (84)	0.007	54.6 (77)	50.4 (71)	0.474
WC (cm)	87 ± 14	95 ± 16	<0.001	92 ± 14	93 ± 14	0.551
Metabolic risk factors						
Fasting glucose (mmol/L)	4.74 (4.38, 5.17)	4.89 (4.62, 5.31)	0.005	4.90 (4.50, 5.32)	4.85 (4.61, 5.21)	0.990
2 h glucose (mmol/L)	6.98 (5.44, 8.67)	7.19 (5.95, 8.63)	0.288	7.31 (5.61, 9.07)	7.05 (5.88, 8.58)	0.096
Fasting insulin (uIU/mL)	10.92 (6.13, 18.44)	11.49 (8.28, 16.19)	0.378	12.55 (7.61, 19.86)	11.20 (8.17, 16.75)	0.326
2 h insulin (uIU/mL)	79.99 (40.68, 154.11)	76.92 (44.55, 123.58)	0.341	97.73 (49.54, 171.86)	76.92 (44.24, 119.14)	0.006
HOMA-IR	2.28 (1.27, 3.90)	2.56 (1.77, 3.77)	0.151	2.86 (1.52, 4.29)	2.44 (1.61, 3.64)	0.326
Matsuda index	3.24 (1.75, 6.49)	3.19 (1.89, 4.92)	0.821	2.55 (1.48, 4.52)	3.29 (2.01, 5.24)	0.020
TG (mmol/L)	1.20 (0.80, 1.80)	1.31 (0.88, 2.04)	0.044	1.30 (0.90, 2.08)	1.26 (0.88, 2.02)	0.952
TC (mmol/L)	4.79 ± 0.91	4.92 ± 0.83	0.158	4.84 ± 0.89	4.90 ± 0.80	0.608
HDL (mmol/L)	1.28 ± 0.36	1.26 ± 0.30	0.643	1.23 ± 0.33	1.28 ± 0.31	0.280
LDL (mmol/L)	2.86 ± 0.82	3.00 ± 0.72	0.087	2.91 ± 0.82	2.97 ± 0.70	0.529
Cardiometabolic risk score (z)	−0.07 ± 0.71	0.08 ± 0.58	0.088	0.13 ± 0.67	0.03 ± 0.56	0.236
Reproductive hormone						
LH (IU/L)	8.87 (5.28, 13.97)	4.14 (2.79, 7.88)	<0.001	7.96 (5.28, 13.38)	4.35 (2.85, 8.44)	<0.001
FSH (IU/L)	6.47 ± 2.20	6.06 ± 3.05	0.207	6.56 ± 2.22	6.14 ± 2.79	0.224
LH/FSH	1.45 (0.88, 2.19)	0.76 (0.56, 1.30)	<0.001	1.45 (0.80, 2.09)	0.78 (0.56, 1.33)	<0.001
T (nmol/L)	2.56 ± 0.94	1.77 ± 0.65	<0.001	2.52 ± 0.94	1.81 ± 0.63	<0.001
FAI	10.22 (6.38, 14.94)	5.36 (2.44, 9.36)	<0.001	10.76 (7.25, 14.69)	5.58 (2.42, 8.44)	<0.001
SHBG (nmol/L)	22.86 (17.17, 34.90)	30.43 (17.34, 43.61)	0.028	22.30 (17.49, 31.85)	30.50 (17.73, 45.07)	0.012
DHEAS (ng/mL)	266.93 ± 114.28	229.95 ± 98.40	0.006	259.06 ± 107.98	234.91 ± 98.32	0.093
A2 (ng/mL)	3.32 (2.66, 4.33)	2.40 (1.82, 3.12)	<0.001	3.35 (2.50, 4.33)	2.43 (1.93, 3.15)	<0.001
Body composite indices						
Visceral Fat Area (cm^2^)	122.80 ± 54.69	146.14 ± 51.39	<0.001	138.31 ± 54.48	142.95 ± 51.54	0.463
Percent Body Fat (%)	35.66 ± 7.56	39.04 ± 6.82	<0.001	37.74 ± 7.02	38.65 ± 6.81	0.271
Trunk BFM/Ht^2^ (kg/m^2^)	4.81 ± 1.95	5.67 ± 1.83	<0.001	5.43 ± 1.92	5.53 ± 1.79	0.661
SMM/Ht^2^ (kg/m^2^)	9.17 ± 1.26	9.55 ± 1.22	0.003	9.55 ± 1.27	9.42 ± 1.11	0.368
SMI (kg/m^2^)	6.93 ± 0.98	7.23 ± 0.97	<0.001	7.21 ± 0.99	7.13 ± 0.88	0.463

Data are mean ± SD, or median (IQR). PSM, propensity score-matched; TG, triglyceride; TC, total cholesterol; LDL, low-density lipoprotein; HDL, high-density lipoprotein; LH, luteinizing hormone; FSH, follicle-stimulating hormone; DHEAS, sulfated dehydroepiandrosterone; T, testosterone; A2, androstenedione; FAI, free androgen index; SHBG, sex hormone-binding globulin; BFM, body fat mass; SMM, skeletal muscle mass; SMI, skeletal muscle index = appendicular muscle mass/Ht^2^. The outcome of cardiometabolic risk is indicated by cardiometabolic risk score, which was derived by standardizing and then summing continuously distributed cardiometabolic risk factors of the z scores of these variables, then dividing them by the number of risk factors to compile the cardiometabolic risk score with units of SD. *p* value ^(1)^: PCOS vs. non-PCOS, ^(2)^ PCOS vs. non-PCOS after PSM.

**Table 2:** Adjusted odds ratios of higher cardiometabolic risk among PCOS and controls by BMI categories.

	PCOS		Control	
Overall	(*n* = 245)	*p* value	(*n* = 156)	*p* value
Trunk BFM/Ht^2^				
unadjusted	2.26 (1.83, 2.79)	<0.001	1.95 (1.49, 2.56)	<0.001
adjusted	2.33 (1.49, 3.65)	0.006	2.02 (1.19, 3.43)	0.009
SMM/Ht^2^				
unadjusted	3.22 (2.34, 4.43)	<0.001	2.07 (1.43, 2.99)	<0.001
adjusted	2.05 (1.12, 3.76)	0.020	1.09 (0.55, 2.18)	0.805
BMI ≥ 28 kg/m^2^	(*n* = 98)		(*n* = 84)	
Trunk BFM/Ht^2^				
unadjusted	1.53 (1.01, 2.31)	0.046	1.58 (1.03, 2.43)	0.036
adjusted	1.36 (0.77, 2.41)	0.293	1.07 (0.49, 2.34)	0.858
SMM/Ht^2^				
unadjusted	2.44 (1.36, 4.36)	0.003	1.68 (1.01, 2.79)	0.047
adjusted	2.27 (1.15, 4.47)	0.018	1.51 (0.69, 3.28)	0.301
BMI < 28 kg/m^2^	(*n* = 147)		(*n* = 72)	
Trunk BFM/Ht^2^				
unadjusted	3.04 (2.02, 4.59)	<0.001	2.72 (1.84, 4.02)	<0.001
adjusted	2.12 (1.06, 4.24)	0.034	2.04 (1.04, 4.02)	0.039
SMM/Ht^2^				
unadjusted	3.28 (1.96, 5.50)	<0.001	3.24 (1.95, 5.40)	<0.001
adjusted	1.64 (0.69, 3.92)	0.267	1.98 (0.84, 4.67)	0.118
*p*-value for interaction *				
BMI * Trunk BFM/Ht^2^	<0.001		<0.001	
BMI * SMM/Ht^2^	<0.001		<0.001	

Higher cardiometabolic risk was analyzed among overall and BMI-stratified PCOS or control women. Adjusted, adjust age, body weight, serum creatinine and γ-glutamyl transferase and trunk BFM/Ht^2^ or SMM/Ht^2^ accordingly. BFM, body fat mass; SMM, skeletal muscle mass. * The interactions of BMI category with trunk BFM/Ht^2^ and SMM/Ht^2^ on cardiometabolic risk was examined by including a respective multiplicative interaction term in the model.

**Table 3:** Cardiometabolic risk factors and reproductive profiles in obese women (BMI ≥ 28 kg/m^2^) among PCOS and control with HA.

	PCOS (*n* = 77)	Control (*n* = 26)	*p* Value
BMI (kg/m^2^)	32.4 (30.1, 34.8)	33.5 (31.1, 37.8)	0.109
WC (cm)	100 (90, 103)	108 (96, 115)	0.019
Fasting glucose (mmol/L)	5.09 (4.64, 5.51)	5.10 (4.70, 5.58)	0.651
2 h glucose (mmol/L)	7.95 (6.66, 9.50)	8.47 (6.90, 10.13)	0.413
Fasting insulin (uIU/mL)	18.60 (13.60, 24. 23)	14.80 (11.49, 19.90)	0.130
2 h insulin (uIU/mL)	129.26 (78.29, 251.78)	135.9 (75.66, 158.75)	0.456
TG (mmol/L)	1.60 (1.06, 2.30)	1.91 (1.45, 2.65)	0.114
HDL (mmol/L)	1.08 (0.98, 1.25)	1.13 (1.05, 1.27)	0.272
LDL (mmol/L)	3.04 (2.63, 3.67)	3.15 (2.70, 4.00)	0.350
Cardiometabolic risk score	0.49 (0.26, 0.83)	0.43 (0.31, 0.80)	0.754
LH (IU/L)	7.96 (5.43, 11.46)	5.33 (3.59, 7.50)	0.013
LH/FSH	1.39 (0.96, 1.65)	1.00 (0.67, 1.52)	0.122
T (nmol/L)	2.53 (2.08, 2.97)	2.38 (1.70, 2.80)	0.122
FAI	13.95 (10.58, 18.25)	14.80 (9.92, 23.80)	0.325
SHBG (nmol/L)	17.95 (12.08, 23.58)	13.79 (10.50, 16.96)	0.006
DHEAS (ng/mL)	242.23 (189.00, 306.64)	295.37 (228.84, 374.37)	0.064
A2 (ng/mL)	3.50 (2.78, 4.32)	3.49 (6.37, 4.40)	0.510
Trunk BFM/Ht^2^ (kg/m^2^)	6.86 (6.00, 7.75)	7.10 (6.37, 8.13)	0.182
SMM/Ht^2^ (kg/m^2^)	10.74 (9.83, 11.36)	10.21 (9.72, 10.78)	0.051

Data are shown as medians (IQR). Wilcoxon rank-sum tests were used to compare differences between PCOS and controls. *p* values less than 0.05 were considered statistically significant. HA, hyperandrogenemia; TG, triglyceride; LDL, low-density lipoprotein; HDL, high-density lipoprotein; LH, luteinizing hormone; DHEAS, sulfated dehydroepiandrosterone; T, testosterone; A2, androstenedione; FAI, free androgen index; SHBG, sex hormone-binding globulin; BFM, body fat mass; SMM, skeletal muscle mass. The outcome of cardiometabolic risk is indicated by cardiometabolic risk score, which was derived by standardizing and then summing continuously distributed cardiometabolic risk factors of the z scores of these variables, then dividing them by the number of risk factors to compile the cardiometabolic risk score with units of SD.

**Table 4:** Clinical characteristics of PCOS subtypes and control women after PSM.

	Metabolic PCOS (*n* = 57)	Control (*n* = 57)	*p* Value ^(1)^	Reproductive PCOS (*n* = 48)	Control (*n* = 48)	*p* Value ^(2)^
Age (years)	28 ± 7	27 ± 6	0.299	29 ± 5	29 ± 6	0.692
BMI (kg/m^2^)	30.1 ± 4.8	28.3 ± 7.7	0.084	24.8 ± 4.56	25.0 ± 4.6	0.815
WC (cm)	100 ± 14	94 ± 22	0.240	84 ± 14	85 ± 15	0.688
Metabolic variables						
Fasting glucose (mmol/L)	4.96 (4.36, 5.40)	4.71 (4.54, 5.19)	0.304	4.60 (4.16, 4.95)	4.81 (4.45, 5.15)	0.029
2 h glucose (mmol/L)	7.46 (6.18, 8.79)	6.78 (5.45, 7.97)	0.066	5.83 (4.74, 7.47)	6.85 (5.47, 8.21)	0.095
Fasting insulin (uIU/mL)	19.25 (13.64, 24.36)	10.82 (6.32, 14.80)	<0.001	6.50 (4.17, 11.77)	8.26 (5.21, 12.76)	0.259
2 h insulin (uIU/mL)	145.16 (102.05, 233.48)	58.93 (38.19, 96.48)	<0.001	51.51 (25.83, 105.76)	57.66 (37.44, 92.32)	0.412
HOMA-IR	4.20 (3.20, 5.36)	2.56 (1.35, 3.50)	<0.001	1.36 (0.88, 2.35)	1.73 (1.05, 2.86)	0.159
Matsuda index	1.83 (1.20, 2.53)	3.53 (2.26, 5.97)	<0.001	5.99 (2.92, 9.92)	4.08 (2.56, 7.73)	0.145
TG (mmol/L)	1.40 (1.06, 1.90)	1.05 (0.80, 1.83)	0.015	1.00 (0.78, 1.60)	1.00 (0.80, 1.92)	0.679
TC (mmol/L)	5.03 ± 0.82	4.77 ± 0.80	0.123	4.88 ± 0.93	4.81 ± 0.76	0.690
HDL (mmol/L)	1.10 ± 0.20	1.30 ± 0.31	<0.001	1.41 ± 0.40	1.30 ± 0.28	0.137
LDL (mmol/L)	3.14 ± 0.73	2.84 ± 0.70	0.039	2.85 ± 0.76	2.86 ± 0.69	0.943
Cardiometabolic risk score	0.39 ± 0.41	−0.15 ± 0.58	<0.001	−0.40 ± 0.64	−0.20 ± 0.53	0.109
Reproductive hormone						
LH (IU/L)	8.72 (7.00, 11.75)	4.14 (3.14, 7.52)	<0.001	12.35 (8.31, 18.70)	4.11 (2.88, 7.10)	<0.001
FSH (IU/L)	5.90 ± 1.65	5.16 ± 2.20	0.517	7.25 ± 1.82	5.97 ± 2.71	0.012
T (nmol/L)	2.71 (2.22, 3.08)	2.05 (1.40, 2.46)	<0.001	2.24 (1.74, 2.24)	1.90 ± 0.66	0.002
FAI	14.66 (11.82, 18.89)	4.48 (2.44, 9.88)	<0.001	7.03 (4.82, 9.28)	3.28 (0.63, 6.28)	<0.001
SHBG (nmol/L)	18.05 (12.93, 21.87)	35.23 (17.32, 56.51)	<0.001	32.53 (22.46, 52.53)	39.14 (27.06, 60.52)	0.152
DHEAS (ng/mL)	293.65 ± 127.04	261.39 ± 98.07	0.193	229.52 ± 118.15	241.07 ± 93.88	0.648
A2 (ng/mL)	3.78 (3.12, 4.60)	2.68 (2.35, 3.64)	<0.001	3.99(2.97, 4.94)	2.65 (2.14, 3.42)	<0.001
Body composite indices						
Trunk BFM/Ht^2^ (kg/m^2^)	6.10 ± 1.57	5.29 ± 2.31	0.032	4.32 ± 1.78	4.33 ± 1.66	0.968
SMM/Ht^2^ (kg/m^2^)	9.98 ± 1.06	9.39 ± 1.38	0.012	8.70 ± 1.02	8.80 ± 0.94	0.613

Data are mean ± SD, or median (IQR). TG, triglyceride; TC, total cholesterol; LDL, low-density lipoprotein; HDL, high-density lipoprotein; LH, luteinizing hormone; FSH, follicle-stimulating hormone; DHEAS, sulfated dehydroepiandrosterone; T, testosterone; A2, androstenedione; FAI, free androgen index; SHBG, sex hormone-binding globulin. The outcome of cardiometabolic risk is indicated by cardiometabolic risk score, which was derived by standardizing and then summing continuously distributed cardiometabolic risk factors of the z scores of these variables, then dividing them by the number of risk factors to compile the cardiometabolic risk score with units of SD. *p* value ^(1)^: metabolic PCOS vs. non-PCOS after PSM, ^(2)^ reproductive PCOS vs. non-PCOS after PSM.

**Table 5:** ORs of body composition for higher cardiometabolic risk in the PCOS subtype.

	Metabolic PCOS		Reproductive PCOS	
	OR (95% CI)	*p* value	OR (95% CI)	*p* value
Trunk BFM/Ht^2^				
model 1	1.67 (1.14, 2.44)	0.008	2.34 (1.54, 3.57)	<0.001
model 2	1.24 (0.77, 2.00)	0.381	2.37 (1.33, 4.21)	0.003
SMM/Ht^2^				
model 1	3.14 (1.52, 6.49)	0.002	2.77 (1.50, 5.11)	<0.001
model 2	2.63 (1.13, 6.16)	0.026	1.31 (0.59, 2.90)	0.513

Model 1, unadjusted; model 2, adjusted for age, body weight, serum creatinine and γ-glutamyl transferase, trunk BFM/Ht^2^ or SMM/Ht^2^. BFM, body fat mass; SMM, skeletal muscle mass.

## Data Availability

Datasets are available on reasonable request to corresponding author.

## Supplementary Materials

The following supporting information can be downloaded at: https://www.mdpi.com/article/10.3390/jcm13020483/s1, Figure S1: Hierarchical clustering of PCOS cases in this study; Figure S2: Clinical trait distribution in PCOS subtypes; Figure S3: PCA plot of traits in PCOS subtypes; Table S1: Association of body composite indices with cardiometabolic risk; Table S2: Body composite tertile range in specific group; Table S3: Cardiometabolic risk factors and reproductive profiles by trunk BFM/Ht2 tertiles; Table S4: Cardiometabolic risk factors and reproductive profiles by SMM/Ht2 tertiles; Table S5: Clinical characteristics of PCOS subtypes and control women before PSM.
