# Supplementary material for: Association of Fat Mass and Skeletal Muscle Mass with Cardiometabolic Risk Varied in Distinct PCOS Subtypes: A Propensity Score-Matched Case-Control Study
Source: J Clin Med. 2024 Jan 15;13(2):483. doi: 10.3390/jcm13020483 (PMC10817046; doi:10.3390/jcm13020483)
Supplement: Supplementary file 1 [file jcm-13-00483-s001.zip › jcm-2771748-supplementary.pdf]

Figure S1. Hierarchical clustering of PCOS cases in this study.

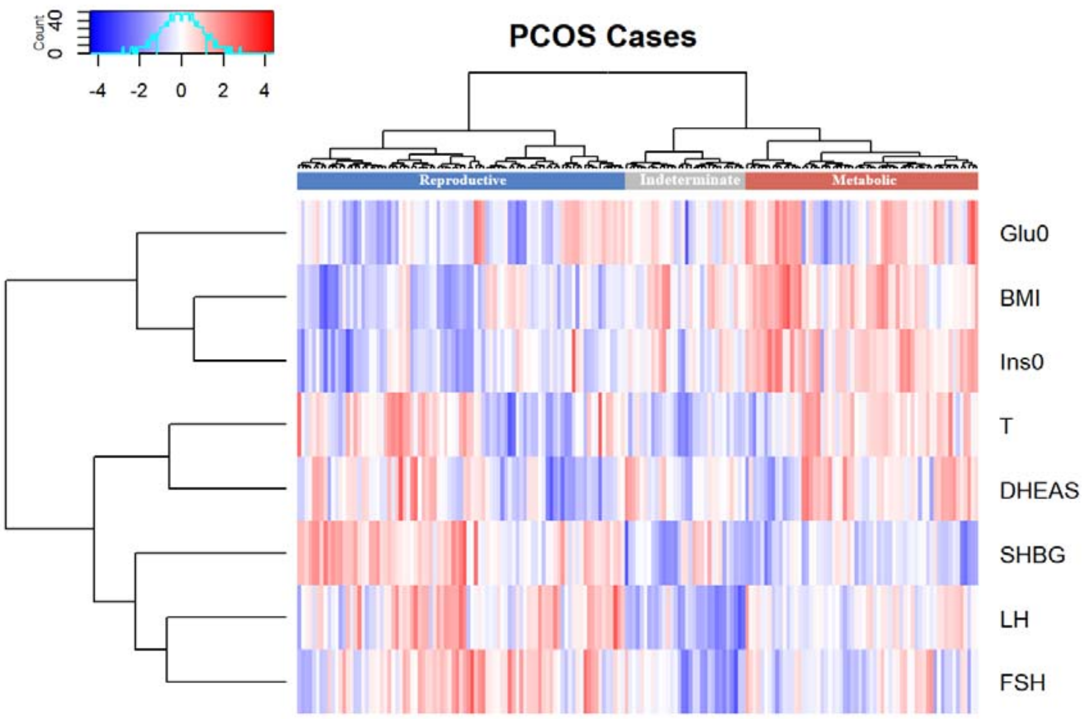

Heatmap from an unsupervised, agglomerative, hierarchical clustering of PCOS. PCOS individuals with complete 8 adjusted quantitative traits: BMI, T, SHBG, DHEAS, fasting glucose and insulin, fasting, LH, and FSH were then subtyped into three subtypes. Each trait was log normalized and adjusted for age and assay method. Ins 0: fasting insulin; BMI, body mass index; Glu0, fasting glucose; DHEAS, sulfated dehydroepiandrosterone; T, testosterone;LH, luteinizing hormone; FSH, follicle stimulating hormone; SHBG, sex hormone binding globulin.Red, metabolic group; Blue, reproductive group; Grey, indeterminate group.

Figure S2. Clinical traits distribution in PCOS subtypes.

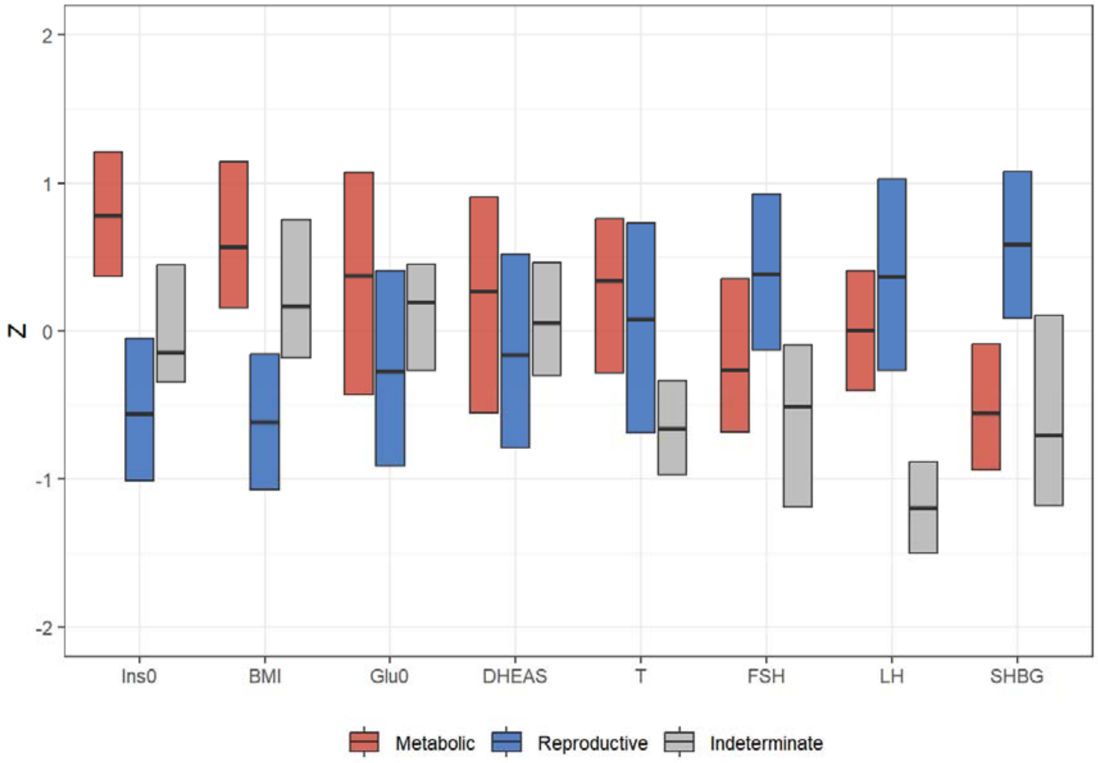

Median and IQRs of normalized adjusted trait in PCOS subtypes are shown. Ins 0: fasting insulin; BMI, body mass index; Glu0, fasting glucose; DHEAS, sulfated dehydroepiandrosterone; T, testosterone;LH, luteinizing hormone; FSH, follicle stimulating hormone; SHBG, sex hormone binding globulin

Figure S3. PCA plot of traits in PCOS subtypes

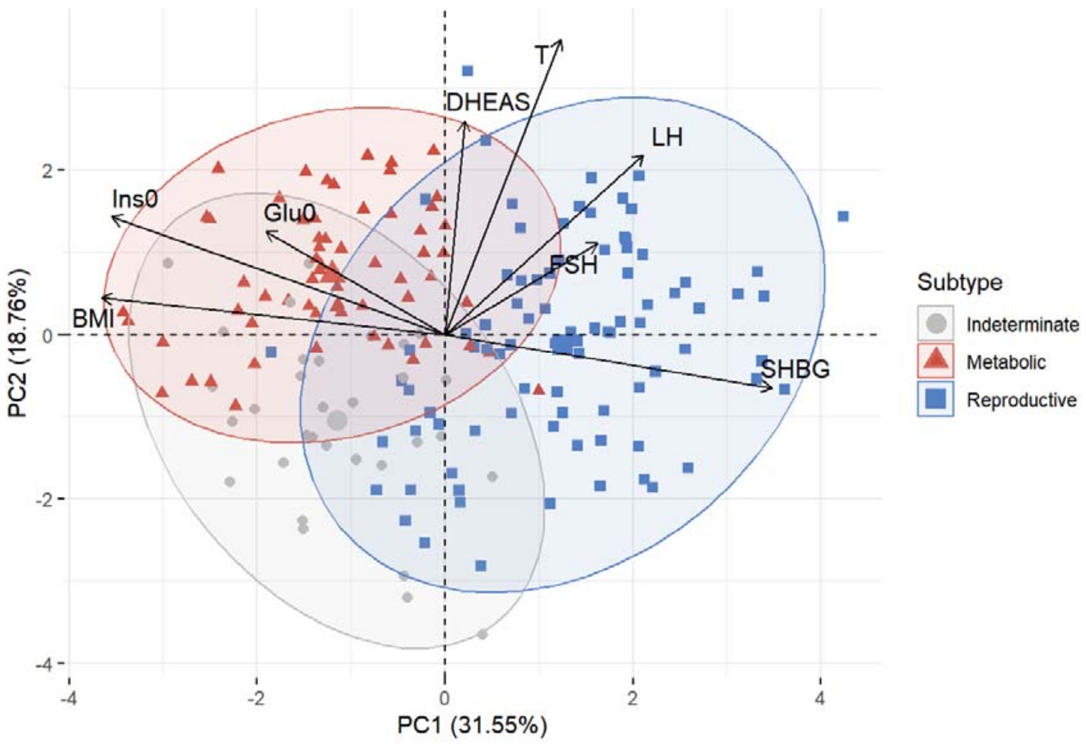

PCOS with complete quantitative trait data were plotted on the first 2 PCs. The relative magnitude and direction of trait correlation with the PCs are shown with black arrows. Ins 0: fasting insulin; BMI, body mass index; Glu0, fasting glucose; DHEAS, sulfated dehydroepiandrosterone; T, testosterone;LH, luteinizing hormone; FSH, follicle stimulating hormone; SHBG, sex hormone binding globulin.

Table S1. Association of body composite indices with cardiometabolic risk

	All		PCOS		Control	
Variables	R^2	P	R^2	P	R^2	P
Fat						
PBF(%)	0.371	<0.001	0.353	<0.001	0.391	<0.001
VFA (cm^2)	0.401	<0.001	0.387	<0.001	0.416	<0.001
BFM(kg)	0.400	<0.001	0.397	<0.001	0.405	<0.001
BFM/Ht^2(kg/m^2)	0.416	<0.001	0.418	<0.001	0.416	<0.001
BFM/BMI (m^2)	0.332	<0.001	0.312	<0.001	0.354	<0.001
Appendicular BFM (kg)	0.347	<0.001	0.359	<0.001	0.342	<0.001
Appendicular BFM/Ht^2(kg/m^2)	0.359	<0.001	0.380	<0.001	0.345	<0.001
Appendicular BFM/BMI(m^2)	0.279	<0.001	0.264	<0.001	0.295	<0.001
Trunk BFM(kg)	0.436	<0.001	0.423	<0.001	0.452	<0.001
**Trunk BFM/Ht^2(kg/m^2)**	**0.458**	**<0.001**	**0.448**	**<0.001**	**0.470**	**<0.001**
Trunk BFM/BMI(m^2)	0.352	<0.001	0.341	<0.001	0.349	<0.001
Muscle						
SMM(kg)	0.275	<0.001	0.279	<0.001	0.252	<0.001
**SMM/Ht^2(kg/m^2)**	**0.395**	**<0.001**	**0.395**	**<0.001**	**0.357**	**<0.001**
SMM/BMI(m^2)	0.207	<0.001	0.207	<0.001	0.218	<0.001
SMM appendicular(kg)	0.237	<0.001	0.237	<0.001	0.215	<0.001
SMM appendicular/Ht^2(kg/m^2)	0.374	<0.001	0.374	<0.001	0.328	<0.001
SMM appendicular/BMI(m2)	0.180	<0.001	0.180	<0.001	0.191	0.004
SMM Trunk(kg)	0.210	<0.001	0.210	<0.001	0.203	<0.001
SMM Trunk/Ht^2(kg/m^2)	0.211	<0.001	0.211	<0.001	0.190	<0.001
SMM Trunk/BMI(m^2)	0.126	<0.001	0.126	<0.001	0.130	<0.001

Body composite indices represent fat or muscle adjusted by $height^2$ or BMI were analysed. The outcome of cardiometabolic risk is indicated by cardiometabolic risk score, which was derived by standardizing and then summing continuously distributed cardiometabolic risk factors of the z scores of these variables, then divided by number of risk factors to compile the cardiometabolic risk score with units of SD. The proportion of the variance explained (R^2) of cardiometabolic risk was caculated in specified group. The best performing body composite indice of fat or muscle (bold) was used in the analysis in our study.VFA,visceral fat area;PBF, percent of body fat; BFM,body fat mass;Ht, height; SMM,skeletal muscle mass.

Table S2.Body composite tertile range in specific group

	BFM Trunk/Ht^2			SMM/Ht^2		
	Tertitle 1	Tertitle 2	Tertitle 3	Tertitle 1	Tertitle 2	Tertitle 3
Overall						
PCOS(N=245)	0.92-3.74	3.75-5.83	5.84-10.07	6.73-8.39	8.40-9.77	9.78-13.27
control (N=156)	1.11-4.86	4.87-6.48	6.49-10.71	6.71-8.95	8.96-9.93	9.94-13.77
BMI≥28Kg/m^2						
PCOS(N=98)	4.38-6.25	6.26-7.24	7.25-10.07	8.21-9.90	9.91-10.50	10.51-13.27
nonPCOS(N=84)	5.02-6.38	6.39-7.35	7.36-10.71	8.52-9.73	9.74-10.64	10.65-13.77
BMI<28Kg/m2						
PCOS(N=147)	0.92-2.96	2.97-4.10	4.11-5.98	6.73-8.00	8.01-8.83	8.84-10.83
nonPCOS(N=72)	1.11-3.95	3.96-4.77	4.78-6.24	6.71-8.40	8.41-9.03	9.04-10.29

Group-specific tertiles of BFM trunk adjusted by height square (BFM Trunk/Ht^2) and skeletal muscle mass adjusted by height square (SMM/Ht^2) were defined among the overall participants and among participants with or without PCOS stratified by BMI, separately.BFM,Body fat mass; SMM, skeletal muscle mass.

Table S3.Cardiometabolic risk factors and reproductive profiles by Trunk BFM /Ht^2 tertiles

	PCOS				p value (tertitle 3 vs. tertile 1)	control				p value (tertitle 3 vs. tertile 1)
	Tertitle 1	Tertitle 2	Tertitle 3	p for trend		Tertitle 1	Tertitle 2	Tertitle 3	p for trend	
**Overall**										
BMI(kg/m^2)	20.6±1.9	26.3±2.3	32.8±2.8	**<0.001**	**<0.001**	23.6±2.8	28.8±1.9	34.4±3.4	**<0.001**	**<0.001**
WC(cm)	75±10	89±12	97±11	**<0.001**	**<0.001**	84±12	93±8	108±7	**<0.001**	**<0.001**
Fasting glocuse (mmc	4.58(4.24, 4.85)	4.76(4.40,5.12)	4.93(4.51,5.45)	**<0.001**	**<0.001**	4.78 (4.44, 4.96)	5.02（4.67, 5.40)	4.96 (4.68, 5.44)	**0.011**	**0.022**
2h glucose(mmol/L)	5.72(4.89,8.20）	6.56(5.43,7.97)	7.69 (76.56,9.35)	**<0.001**	**<0.001**	6.42 (5.32, 8.27)	7.12 (5.81, 8.74)	7.87 (6.71,9.08)	**0.006**	**0.010**
Fasting insulin(uIU/m	4.84（3.54,10.25)	10.92(7.52,15.71)	18.40(12.92, 23.76)	**<0.001**	**<0.001**	8.25 (4.84, 10.46)	11.65 (8.83,14.35)	14.81 (11.34, 20.68)	**<0.001**	**<0.001**
2h insulin(uIU/ml)	48.81(27.30,93.20)	76.40(39.20,132.9	136.05(81.18,240.5	**<0.001**	**<0.001**	52.38 (26.95, 90.30)	75.42 (47.72, 122.44	92.12 (58.20,149.78)	**<0.001**	**<0.001**
TG(mmol/L)	0.80(0.60,1.22)	1.29(0.92,1.69)	1.50(1.00, 2.10)	**<0.001**	**<0.001**	1.00 (0.80, 1.83)	1.46 (1.07, 2.18)	1.64 (0.88, 2.17)	**0.006**	**0.027**
HDL(mmol/L)	1.53±0.36	1.21±0.29	1.14±0.21	**<0.001**	**<0.001**	1.39±0.32	1.21±0.30	1.24±0.24	**0.010**	**0.016**
LDL(mmol/L)	2.61±0.69	2.86±0.74	3.10±0.76	**<0.001**	**<0.001**	2.80±0.66	3.06±0.73	3.08±0.68	0.116	0.061
Cardiometabolic risk score	-0.66±0.56	-0.05±0.49	0.42±0.51	**<0.001**	**<0.001**	-0.35±0.49	0.11±0.41	0.36±0.39	**<0.001**	**<0.001**
LH(IU/L)	10.45(6.17,18.53)	9.67(4.70,15.79)	7.38(4.56,10.44)	**0.002**	**<0.001**	3.73 (2.63, 8.61)	5.33 (3.00,7.48)	4.06 (2.73, 8.92)	0.869	0.877
LH/FSH	1.83(0.94,2.79)	1.50(0.98,2.54)	1.23 (0.72,1.61)	**0.002**	**0.001**	0.72 (0.55,1.27)	0.87 (0.54,1.22)	0.95 (0.57, 1.67)	0.479	0.403
T(nmol/L)	2.72±0.93	2.45±0.81	2.49±0.92	0.160	0.139	1.70±0.63	1.72±0.66	1.89±0.68	0.414	0.217
FAI	7.34(4.90, 11.56)	10.51(7.26,17.28)	12.84(8.72,17.14)	**<0.001**	**<0.001**	3.01 (1.21, 5.44）	6.56 (4.44,14.12)	7.34 (4.14, 16.36)	**<0.001**	**<0.001**
SHBG(nmol/L)	37.08(20.38, 37.08)	21.78(15.30,33.30	19.95(14.17,25.60)	**<0.001**	**<0.001**	41.55（31.40, 5.44)	21.27 (15.73, 39.02)	19.55 (13.80,31.42)	**<0.001**	**<0.001**
DHEAS(ng/ml)	268±96	257±117	263±113	0.836	0.784	228±96	234±114	219±80	0.871	0.695
A2(ng/ml)	3.30(2.70, 4.47)	3.22(2.66,4.14)	3.36 (2.44,4.32)	0.664	0.390	2.53 (2.05,2.95）	2.42(1.81,4.14)	2.16 (1.69, 2.87)	0.654	0.419
Trunk BFM/Ht^2	2.65±0.65	4.21±0.45	7.02±0.84	**<0.001**	**<0.001**	3.76±1.00	4.89±0.45	7.58±0.83	**<0.001**	**<0.001**
SMM/Ht^2(kg/m^2)	8.07±0.77	9.11 (8.43, 9.61)	10.27±0.92	**<0.001**	**<0.001**	8.59±0.82	9.65±0.89	10.40±1.16	**<0.001**	**<0.001**
**BMI≥28Kg/m^2**										
BMI(kg/m^2)	29.5±1.5	32.9±2.7	32.2±2.9	**<0.001**	**<0.001**	29.9±1.3	31.7±1.5	36.7±2.9	**<0.001**	**<0.001**
WC(cm)	96±18	97±11	97±13	0.297	0.228	94±10	102±7	111±5	**<0.001**	**<0.001**
Fasting glocuse (mmc	4.91(4.31,5.62)	4.74(4.38,5.16)	5.25(4.72,5.77)	0.050	0.166	5.11 (4.85, 5.61)	5.02(4.83, 5.38)	4.77 (4.61,5.61)	**0.009**	**0.029**
2h glucose(mmol/L)	7.78(5.65,10.01)	7.30(6.60,9.00)	7.84(6.59,9.71)	0.808	0.649	7.07 (5.70, 8.66)	8.26 (7.32, 9.83)	7.46 (6.42, 8.73)	0.410	0.589
Fasting insulin(uIU/m	13.14(9.03,21.39)	17.59(12.77,23.15	19.85(14.55,28.46)	**0.022**	**0.007**	11.66 (8.89, 13.51)	14.59 (11.30, 19.32)	16.18 (11.94, 27.61)	0.103	**<0.001**
2h insulin(uIU/ml)	78.60(1.50,2.40)	149.46(100.84,245	149.84(93.48,283.0	**0.005**	**0.002**	76.40 (44.55,130.09	102.82 (67.98, 155.1	91.04 (48.46, 153.40	0.077	0.187
TG(mmol/L)	1.02(1.50,2.40)	1.50(1.02,1.99)	1.70(1.16,2.10)	0.576	0.583	1.71 (1.22, 2.34)	1.77 (1.18, 1.91)	1.60 (0.87, 2.73)	0.830	0.678
HDL(mmol/L)	1.12±0.20	1.14±0.21	1.13±0.21	0.748	0.832	1.23±0.33	1.20±0.26	1.25±0.21	0.831	0.823
LDL(mmol/L)	2.90±0.87	3.11±0.76	3.07±0.78	0.371	0.190	3.29±0.68	2.94±0.66	3.12±0.73	0.267	0.430

Cardiometabolic risk score	0.20±0.55	0.44±0.50	0.40±0.52	**0.004**	**0.002**	0.18±0.42	0.39±0.44	0.38±0.44	0.159	0.132
LH(IU/L)	8.72(4.58,11.39)	5.66(3.05,9.66)	7.96(6.92,12.39)	0.051	0.428	4.13 (1.72, 6.21)	4.08 (2.21,9.94)	4.51 (2.91, 8.48)	0.364	0.164
LH/FSH	1.37(0.89,1.68)	0.97(0.62,1.57)	1.46(1.09,1.88)	0.053	0.385	0.71 (0.40, 1.20)	0.96 (0.51, 2.13)	0.99 (0.63, 1.36)	0.304	0.071
T(nmol/L)	2.23±0.60	2.48±0.93	2.43±0.88	0.614	0.368	1.56±0.64	1.78±0.56	1.99±0.80	0.166	0.084
FAI	12.82(6.40,17.77)	11.19(6.73,14.63)	14.31(9.31,21.58)	0.212	0.243	6.56 (4.64, 14.11)	6.23 (5.30, 12.37)	9.30 (3.80, 25.50)	**0.017**	0.662
SHBG(nmol/L)	21.0(12.05,30.00)	19.50(17.49,28.80	18.57(12.46,24.95)	0.559	0.412	19.94 (14.29, 37.21)	19.54 (15.66, 32.37)	17.78 (11.87, 32.14)	**0.010**	0.813
DHEAS(ng/ml)	97±24	114±14	112±12	0.837	0.603	224±104	203±76	221±98	0.836	0.952
A2(ng/ml)	2.91(2.21,3.95)	3.04(2.06,3.71)	3.69(2.97,4.60)	**0.024**	**0.021**	2.38 (1.81, 4.10)	2.26 (1.61, 2.62)	2.15 (1.80, 3.50)	**0.048**	0.663
Trunk BFM/Ht^2	5.68±0.46	6.52±0.65	7.92±0.42	**<0.001**	**<0.001**	5.79±0.35	6.98±0.72	8.19±0.67	**<0.001**	**<0.001**
SMM/Ht^2(kg/m^2)	9.99±0.82	10.16±0.72	10.69±1.01	**0.004**	**0.018**	10.03±0.69	9.87±0.85	10.93±1.19	**<0.001**	**0.043**
**BMI<28Kg/m^2**										
BMI(kg/m^2)	19.6±1.3	23.0±1.6	25.9±1.4	**<0.001**	**<0.001**	21.1±2.3	25.3±1.3	27.0±0.8	**<0.001**	**<0.001**
WC(cm)	71±6	79±11	88±7	**<0.001**	**<0.001**	74±9	90±10	90±7	**<0.001**	**<0.001**
Fasting glocuse (mmo	4.47(2.24,4.68)	4.75(4.40,5.07)	4.75（4.33,5.09）	**0.009**	**0.015**	4.63 (4.30,4.90)	4.79 (4.45, 5.00)	4.87 (4.59, 5.43)	0.388	0.190
2h glucose(mmol/L)	5.42(4.57,6.79)	7.23(5.27,10.70)	6.48（5.57,8.07）	**<0.001**	**<0.001**	5.45 (4.96, 6.55)	6.62 (5.92, 8.28)	7.20 (5.83, 8.59)	0.056	**0.043**
Fasting insulin(uIU/m	4.18(3.25,8.20)	7.98(5.75,10.70)	11.68（7.72,19.66）	**<0.001**	**<0.001**	5.65 (3.91, 8.42)	8.96 (6.05, 12.77)	9.85 (8.27, 14.27)	**0.001**	**<0.001**
2h insulin(uIU/ml)	42.19(20.76,76.66)	55.41(37.97,82.96	104.01（50.39,137.	**<0.001**	**<0.001**	29.59 (22.88, 53.28)	80.12 (40.88, 115.89	60.15 (47.57, 108.55	**0.002**	**<0.001**
TG(mmol/L)	0.70(0.60,1.00)	1.10(0.80,1.30)	1.23(0.90,1.82)	**<0.001**	**<0.001**	0.84 (0.74, 1.40)	1.01 (0.80, 1.98)	1.38 (0.97, 2.04)	0.109	**0.041**
HDL(mmol/L)	1.65±0.33	1.35±0.34	1.18±0.27	**<0.001**	**<0.001**	1.47±0.33	1.37±0.31	1.19±0.27	**0.018**	**0.006**
LDL(mmol/L)	2.51±0.65	2.83±0.71	2.83±0.71	0.050	**0.031**	2.56±0.62	2.92±0.64	2.97±0.71	0.122	0.065
Cardiometabolic risk score	-0.87±0.48	-0.32±0.43	-0.03±0.45	**<0.001**	**<0.001**	-0.69±0.38	0.17±0.41	-0.01±0.33	**<0.001**	**<0.001**
LH(IU/L)	10.11(5.35,19.89)	12.50(6.86,16.83)	7.88(3.86,13.84)	0.050	0.060	3.68 (2.81,5.77)	4.11 (2.34, 11.01)	5.95 (4.35,10.42)	0.054	0.051
LH/FSH	1.72(0.88,2.99)	1.92(1.23,2.36)	1.45(0.70,3.02)	0.644	0.611	0.63 (0.52, 0.86)	0.71 (0.57, 1.80)	1.18 (0.70, 1.49)	0.100	**0.028**
T(nmol/L)	2.86±1.00	2.44±0.68	2.56±0.92	0.099	0.153	1.80±0.62	1.65±0.66	1.87±0.63	0.550	0.767
FAI	6.37(3.09,10.57)	8.14(5.74,11.72)	11.4(8.27,19.04)	**<0.001**	**<0.001**	2.35 (0.04, 4.17)	3.36 (1.34, 5.28)	6.35 (4.22, 11.33)	**<0.001**	**0.001**
SHBG(nmol/L)	42.32(28.43,72.63)	27.91（20.00,39.0	20.10(14.20,31.02)	**<0.001**	**<0.001**	53.20 (32.75, 74.44)	42.20 (31.34, 57.30)	25.21 (18.33, 40.54)	**0.001**	**<0.001**
DHEAS(ng/ml)	268±103	260±87	272±127	0.872	0.846	236±94	226±106	250±112	0.832	0.715
A2(ng/ml)	3.35(2.78,4.61)	3.13（2.59,4.40）	3.60(2.70,4.36)	0.702	0.860	2.64 (1.96, 3.29)	2.36 (1.77, 2.77)	2.66 (2.21, 4.34)	0.093	0.132
Trunk BFM/Ht^2	2.67±0.49	3.51±0.40	4.75±0.48	**<0.001**	**<0.001**	2.79±0.86	4.11±0.71	5.28±0.37	**<0.001**	**<0.001**
SMM/Ht^2(kg/m^2)	7.83±0.53	8.51±0.89	8.95±0.70	**<0.001**	**<0.001**	8.10±0.70	8.98±0.69	9.02±0.65	**<0.001**	**<0.001**

Data are mean ±SD for continuous variables with normal distribution, or median (IQR) for continuous variables with skewed distribution. Oneway Analysis of Variance (ANOVA) or Kruskal–Wallis H-Test was applied accordingly. Group-specific tertiles of trunk BFM adjusted by height square (Trunk BFM/Ht^2) were defined among the overall participants and among participants with or without PCOS stratified by BMI, separately.The outcome of cardiometabolic risk is indicated by cardiometabolic risk score, which was derived by standardizing and then summing continuously distributed cardiometabolic risk factors of the z scores of these variables, then divided by number of risk factors to compile the cardiometabolic risk score with units of SD. BFM,Body fat mass.TG, triglyceride;LDL, low-density lipoprotein; HDL, high-density lipoprotein; LH, luteinizing hormone;DHEAS, sulfated dehydroepiandrosterone; T, testosterone; A2,androstenedione; FAI, Free androgen index;SHBG, sex hormone [illegible]

Table S4.Cardiometabolic risk factors and reproductive profiles by SMM/Ht^2 tertiles

	PCOS					control				
	Tertitle 1	Tertitle 2	Tertitle 3	p for trend	p value (tertitle3 vs. tertile1)	Tertitle 1	Tertitle 2	Tertitle 3	p for trend	p value (tertitle 3 vs. tertile1)
Overall										
BMI(kg/m^2)	21.3±2.9	26.5±3.3	32.3±3.5	**<0.001**	**<0.001**	21.3±2.5	25.3±1.3	31.4±3.9	**<0.001**	**<0.001**
WC(cm)	75±8	89±10	96±14	**<0.001**	**<0.001**	75±10	90±10	99±11	**<0.001**	**<0.001**
Fasting glocuse (mmol/l	4.60 (4.34, 4.99)	4.74(4.25, 5.06)	4.99 (4.58, 5.51)	**<0.001**	**<0.001**	4.82 (4.47,5.00)	4.91(4.63, 5.32)	5.05 (4.72, 5.68)	**0.011**	0.050
2h glucose(mmol/L)	5.88 (4.89, 8.31)	6.60 (5.56, 7.91)	8.22 (6.63 , 9.71)	**<0.001**	**<0.001**	6.54(5.45,8.28)	7.00 (5.74, 8.50)	7.80 (6.54, 9.54)	**0.006**	**0.034**
Fasting insulin(uIU/ml)	5.94 (3.87,10.03)	11.31 (7.59, 18.10	18.49 (12.16, 23.07	**<0.001**	**<0.001**	8.42 (5.65, 11.00)	12.44 (8.77,14.88)	14.37 (11.07, 20.96)	**<0.001**	**<0.001**
2h insulin(uIU/ml)	49.29 (28.73, 96.56	70.44 (44.88, 135.6	131.44 (77.90, 228.	**<0.001**	**<0.001**	53.00 (36.50, 87.76	76.66 (45.91, 122.2	106.80 (54.47, 151.0	**<0.001**	**<0.001**
TG(mmol/L)	0.93 (0.61, 1.35)	1.17 (0.78, 1.83)	1.50 (1.10, 2.05)	**<0.001**	**<0.001**	1.20 (0.80,1.99)	1.39 (0.88, 2.22)	1.64 (1.07, 2.05)	**0.006**	0.064
HDL(mmol/L)	1.49±0.37	1.26±0.30	1.11±0.21	**<0.001**	**<0.001**	1.46±0.33	1.37±0.31	1.22±0.27	**0.002**	**0.006**
LDL(mmol/L)	2.61±0.62	2.80±0.76	3.18±0.76	**<0.001**	**<0.001**	2.64±0.70	2.92±0.64	3.07±0.69	**0.048**	0.920
Cardiometabolic risk score	-0.60±0.57	-0.11±0.53	0.46±0.48	**<0.001**	**<0.001**	-0.64±0.43	-0.17±0.41	0.24±0.41	**<0.001**	**<0.001**
LH(IU/L)	12.76 (7.67, 19.86)	7.88 (4.81,12.77)	7.51 (4.93, 10.67)	**0.001**	**<0.001**	4.84 (3.34, 9.41)	4.08 (2.84, 8.95)	3.86 (2.17, 7.46)	0.869	0.126
LH/FSH	2.18(1.26, 3.03)	1.27 (0.74, 2.02)	1.31 (0.89, 1.61)	**<0.001**	**<0.001**	0.76 (0.56,1.45)	0.91 (0.58, 1.38)	0.70 (0.53, 1.22)	0.479	0.208
T(nmol/L)	2.64±0.87	2.57±0.98	2.42±0.79	0.359	0.135	1.78±0.62	1.65±0.66	1.80±0.66	0.655	0.742
FAI	7.69 (4.78, 11.64)	10.42 (7.08,15.38)	13.14 (8.99, 17.84)	**<0.001**	**<0.001**	3.79(1.90, 6.13)	4.76 (2.32, 7.14)	9.60 (5.42, 19.00)	**<0.001**	**<0.001**
SHBG(nmol/L)	35.06 (21.89, 50.13	21.43 (16.84, 30.2	19.40 (13.51, 25.04	**<0.001**	**<0.001**	40.72 (30.27,57.11)	31.20 (18.76, 43.71	17.10 (13.23, 26.55)	**<0.001**	**<0.001**
DHEAS(ng/ml)	266±101	274±121	246±102	0.273	0.242	238±91	226±106	224±98	0.882	0.976
A2(ng/ml)	3.77 (2.99, 4.72)	3.17 (2.51, 4.25)	3.22 (2.37, 4.20)	0.073	0.060	2.62(1.91, 3.32)	2.37 (1.81, 2.67)	2.39 (1.82,3.25)	0.654	0.849
Trunk BFM/Ht^2(kg/m^2)	3.06±1.13	4.85±1.43	6.55±1.46	**<0.001**	**<0.001**	4.18±1.49	5.81±1.22	7.03±1.51	**<0.001**	**<0.001**
SMM/Ht^2(kg/m^2)	7.82±0.39	8.99±0.33	10.57±0.64	**<0.001**	**<0.001**	8.36±0.51	9.24±0.56	10.72±0.58	**<0.001**	**<0.001**
BMI≥28Kg/m^2										
BMI(kg/m^2)	30.5±2.1	32.0±2.1	34.4±2.9	**<0.001**	**<0.001**	30.3±1.9	32.6±2.6	35.0±3.9	**<0.001**	**<0.001**
WC(cm)	92±9	99±8	99±18	0.255	0.185	99±12	99±11	107±8	0.051	**0.034**
Fasting glocuse (mmol/l	4.75 (4.32, 5.21)	5.04 (4.52, 5.69)	5.22 (4.73, 5.51)	**0.047**	**0.016**	5.01 (4.89,5.35)	4.79 (4.61, 5.10)	5.22 (4.76, 6.51)	**0.009**	0.744
2h glucose(mmol/L)	7.00 (5.65, 8.02)	8.83 (7.57, 11.13)	7.86 (6.41, 9.71)	**0.014**	**0.209**	7.93 (6.64, 8.77)	7.19 (6.33,8.69)	8.01 (6.46, 11.14)	0.410	0.948
Fasting insulin(uIU/ml)	14.5 (9.9, 20.01)	17.6 (13.0, 23.8)	19.7 (11.7, 27.5)	0.154	0.068	13.32 (10.17,16.90)	12.31(10.88, 16.01)	17.66 (11.65,27.82)	0.103	0.272
2h insulin(uIU/ml)	104.6 (55.2, 182.6)	141.0 (97.8, 243.8)	130.2 (70.9, 255.6)	0.252	0.167	83.69(63.57, 142.8	84.63 (47.19,130.0	117.23 (59.18, 151.8	0.077	0.530
TG(mmol/L)	1.51 (0.93, 2.10)	1.50 (1.07, 2.11)	1.71 (1.13, 2.10)	0.868	0.675	1.71 (0.88, 2.69)	1.64 (0.88, 2.46)	1.73 (1.18, 2.17)	0.830	0.601
HDL(mmol/L)	1.16±0.24	1.16±0.28	1.08±0.19	0.256	0.177	1.26±0.34	1.21±0.25	1.22±0.22	0.851	0.704
LDL(mmol/L)	2.80±0.73	3.26±0.90	3.19±0.65	**0.042**	**0.030**	3.35±0.60	2.96±0.81	3.07±0.61	0.179	0.153

Cardiometabolic risk score	0.16±0.51	0.51±0.37	0.55±0.57	**0.004**	**0.006**	0.32±0.40	0.17±0.38	0.47±0.40	**0.045**	0.214
LH(IU/L)	7.67 (4.88, 12.00)	9.45 (6.85, 12.56)	6.31 (3.82, 9.95)	0.141	0.473	4.11 (2.80,8.34)	3.04 (2.13, 7.56)	4.98 (2.21,7.64)	0.364	0.515
LH/FSH	1.24 (0.62, 1.67)	1.48 (1.26, 1.68)	1.05 (0.82, 1.60)	0.125	0.856	1.13(0.66, 1.98)	0.67 (0.48, 1.17)	0.89(0.58,1.40)	0.304	0.718
T(nmol/L)	2.46±1.00	2.48±0.91	2.35±0.72	0.846	0.650	1.57±0.50	1.73±0.63	1.98±0.82	0.189	0.068
FAI	11.08 (6.40, 14.69)	15.54 (8.50, 21.92)	12.78 (9.71, 17.41)	0.096	0.234	5.72 (4.36, 9.00)	6.36 (2.18, 13.64)	11.30(5.51, 21.35)	**0.017**	**0.009**
SHBG(nmol/L)	21.15 (17.40, 29.5	17.60 (11.81, 29.24	19.36 (16.59, 22.20	0.350	0.219	25.06 (16.09, 35.93	25.63 (13.23, 41.57	16.27 (12.77, 23.28)	**0.010**	**0.001**
DHEAS(ng/ml)	268±118	267±124	232±91	0.373	0.186	166±83	207±60	268±102	**0.017**	**0.014**
A2(ng/ml)	3.09 (2.43, 3.97)	3.24 (2.37, 4.33)	3.31 (2.72, 4.03)	0.810	0.593	2.16 (1.79,2.63)	1.95 (1.68, 2.39)	2.59 (1.92, 4.77)	**0.048**	0.052
Trunk BFM/Ht^2(kg/m^2)	6.46±0.87	6.58±0.86	7.28±1.30	**0.003**	**0.020**	6.51±0.68	6.79±1.06	7.64±1.46	**<0.001**	**0.038**
SMM/Ht^2(kg/m^2)	9.40±0.44	10.26±0.42	11.17±0.55	**<0.001**	**<0.001**	9.25±0.34	10.01±0.23	11.11±0.49	**<0.001**	**<0.001**
BMI<28Kg/m2										
BMI(kg/m^2)	20.1±1.8	22.9±2.3	25.6±1.7	**<0.001**	**<0.001**	22.0±3.1	24.8±2.0	26.7±1.0	**<0.001**	**<0.001**
WC(cm)	72±8	80±7	88±9	**<0.001**	**<0.001**	78±11	87±5	90±11	**0.002**	**0.004**
Fasting glocuse (mmol/l	4.58 (4.33, 4.81)	4.58 (4.23, 5.00)	4.80 (4.45, 5.09)	0.063	**0.030**	4.66 (4.35, 5.03)	4.81 (4.40, 4.91）	4.81(4.45,5.18)	0.858	0.734
2h glucose(mmol/L)	5.43 (4.69, 7.16)	6.81 (5.50, 8.69)	6.59 (5.47,7.95)	**0.003**	**0.002**	6.50 (5.19, 8.28)	6.47(5.45, 8.19）	6.69 (5.31,8.18)	0.976	0.892
Fasting insulin(uIU/ml)	4.73 (3.48, 8.34)	8.05 (4.59, 11.49)	10.41 (7.64, 18.72)	**<0.001**	**<0.001**	6.82 (4.81, 8.82)	8.74 (5.79, 12.64)	9.94 (8.00, 14.90)	0.063	**0.024**
2h insulin(uIU/ml)	45.65 (19.61, 65.72	79.30 (36.51, 134.2	64.35 (44.56, 136.6	**0.006**	**0.004**	53.00 (29.59, 90.81	47.74(29.23, 80.07)	65.06 (37.04, 108.55	0.280	0.211
TG(mmol/L)	0.80 (0.60,1.20)	1.10 (0.70, 1.51)	1.26 (0.81,1.62)	**0.005**	**<0.001**	1.21 (0.80, 2.05)	1.01(0.80,1.68)	1.05 (0.80,1.41)	0.573	0.264
HDL(mmol/L)	1.58±0.37	1.41±0.33	1.19±0.31	**<0.001**	**<0.001**	1.35±0.28	1.38±0.33	1.28±0.35	0.550	0.459
LDL(mmol/L)	2.50±0.70	2.82±0.61	2.82±0.76	0.054	0.051	2.91±0.80	2.81±0.68	2.78±0.60	0.840	0.568
Cardiometabolic risk score	-0.79±0.50	-0.34±0.55	-0.10±0.46	**<0.001**	**<0.001**	-0.33±0.55	-0.32±0.46	-0.16±0.40	0.411	0.247
LH(IU/L)	14.60 (8.16,20.76)	11.58 (5.41,16.65)	7.67(4.00,12.07)	0.052	**0.016**	4.11 (3.54, 8.62)	5.61 (2.62,8.68)	5.20 (2.76, 10.54)	0.994	0.930
LH/FSH	2.31 (1.23,3.02)	1.93(1.08, 2.90)	1.15 (0.69,2.03)	**0.049**	**0.020**	0.74 (0.55, 1.22)	0.91 (0.59, 1.49)	0.72(0.54, 1.19)	0.678	0.965
T(nmol/L)	2.77±0.91	2.54±0.92	2.57±0.85	0.469	0.183	1.78±0.59	1.87±0.72	1.63±0.58	0.505	0.496
FAI	7.56 (3.70, 11.69)	8.17 (5.13, 10.42)	11.40(7.89, 17.72)	**0.002**	**0.001**	3.21 (0.05, 5.34)	4.44 (2.35,6.62)	4.13(2.01, 7.07)	0.289	0.334
SHBG(nmol/L)	35.73(29.10, 58.96	30.20 (18.45, 43.43	20.70 (16.10, 27.58	**<0.001**	**<0.001**	40.90 (33.19, 64.52	35.00(25.58, 56.42)	31.82 (19.54, 57.64)	0.353	0.188
DHEAS(ng/ml)	262±101	271±114	268±107	0.924	0.789	226±91	266±123	218±87	0.419	0.820
A2(ng/ml)	3.66 (3.00, 4.93)	3.70 (2.74, 4.47)	3.21 (2.43, 4.10)	0.323	0.162	2.77 (1.69, 3.62)	2.58 (2.18, 2.77)	2.40 (2.04, 3.00)	0.709	0.535
Trunk BFM/Ht^2(kg/m^2)	2.69±0.90	3.56±1.09	4.25±0.78	**<0.001**	**<0.001**	3.42±1.33	4.33±0.99	4.71±0.46	**<0.001**	**0.002**
SMM/Ht^2(kg/m^2)	7.55±0.30	8.08±0.26	9.41±0.54	**<0.001**	**<0.001**	7.87±0.41	8.47±0.49	9.52±0.36	**<0.001**	**<0.001**

Data are mean ±SD for continuous variables with normal distribution, or median (IQR) for continuous variables with skewed distribution.Oneway Analysis of Variance (ANOVA) or Kruskal–Wallis H-Test was applied accordingly. Group-specific tertiles of SMM adjusted by height square (SMM/Ht^2) were defined among the overall participants and among participants with or without PCOS stratified by BMI, separately.The outcome of cardiometabolic risk is indicated by cardiometabolic risk score, which was derived by standardizing and then summing continuously distributed cardiometabolic risk factors of the z scores of these variables, then divided by number of risk factors to compile the cardiometabolic risk score with units of SD. SMM, skeletal muscle mass; BFM,Body fat mass; TG, triglyceride; LDL, low-density lipoprotein; HDL, high-density lipoprotein; LH, luteinizing hormone; DHEAS, sulfated dehydroepiandrosterone; T, testosterone; A2,androstenedione; FAI, Free androgen index;SHBG, sex hormone binding globulin.

Table S5.Clinical Characteristics of PCOS subtypes and control women before PSM.

	Metabolic PCOS (N=62)	Reproductive PCOS (N=87)	p value
Age(years)	27±7	27±5	0.684
BMI(kg/m^2)	30.9±4.8	22.8±4.2	<0.001
WC(cm)	99±14	79±12.9	<0.001
**Metabolic variables**			
Fasting glocuse (mmol/L)	4.99(4.37,5.39)	4.58(4.24,4.95)	<0.001
2h glucose(mmol/L)	7.39(6.21,8.83	5.60(4.91,7.32)	<0.001
Fasting insulin(uIU/ml)	18.89(13.98,24.30)	6.20(3.93,10.71)	<0.001
2h insulin(uIU/ml)	141.21(103.33,222.82)	48.91(25.40,92.66)	<0.001
HOMA-IR	4.19(3.27,5.27)	1.33(0.82,2.24)	<0.001
Matsuda index	1.84(1.20,2.52)	6.34(3.50,10.18)	<0.001
TG(mmol/L)	1.45(1.02,1.98)	0.90(0.62,1.35)	<0.001
TC(mmol/L)	5.05±0.83	4.68±0.86	0.012
HDL(mmol/L)	1.13(1.00,1.25)	1.49(1.23,1.81)	<0.001
LDL(mmol/L)	3.05(2.69,3.61)	2.49(2.19,3.04)	<0.001
Cardiometabolic risk score	0.40±0.42	-0.57±0.61	<0.001
**Reproductive hormone**			
LH(IU/L)	8.78(7.03,12.57)	12.63(8.09,19.02)	<0.001
FSH(IU/L)	5.99±1.63	7.24±1.81	<0.001
T(nmol/L)	2.84±0.80	2.69±1.07	0.352
FAI	14.74(12.17,20.78)	7.25(4.87,9.95)	<0.001
SHBG(nmol/L)	17.87(13.01,21.86)	34.25(24.60,54.88)	<0.001
DHEAS(ng/ml)	281.05(196.75,281.05)	230.16(167.72,327.13)	0.03
A2(ng/ml)	3.71(3.03,4.46)	3.58(2.87,4.51)	0.438
**Body composite indices**			
Trunk BFM/Ht^2(kg/m^2)	6.21±1.57	3.60±1.63	<0.001
SMM/Ht^2(kg/m^2)	10.02±1.04	8.29±0.96	<0.001

Data are mean ±SD, or median (IQR).TG, triglyceride; Tch, total cholesterol; LDL, low-density lipoprotein; HDL, high-density lipoprotein; LH, luteinizing hormone; FSH, follicle stimulating hormone; E2, estradiol; DHEAS, sulfated dehydroepiandrosterone; T, testosterone; A2,androstenedione; FAI, Free androgen index;HA, hyperandrogenemia; SHBG, sex hormone binding globulin.The outcome of cardiometabolic risk is indicated by cardiometabolic risk score, which was derived by standardizing and then summing continuously distributed cardiometabolic risk factors of the z scores of these variables, then divided by number of risk factors to compile the cardiometabolic risk score with units of SD.
